# Dynamic measurement of near-field radiative heat transfer

**DOI:** 10.1038/s41598-017-14242-x

**Published:** 2017-10-24

**Authors:** S. Lang, G. Sharma, S. Molesky, P. U. Kränzien, T. Jalas, Z. Jacob, A. Yu. Petrov, M. Eich

**Affiliations:** 1 Institute of Optical and Electronic Materials, Hamburg University of Technology, Eissendorfer Strasse 38, 21073 Hamburg, Germany; 2 University of Alberta, Department of Electrical and Computer Engineering, 9107 - 116 Street, Edmonton, T6G 2V4 Canada; 3 Birck Nanotechnology Center, School of Electrical and Computer Engineering, Purdue University, West Lafayette, IN 47906 USA; 4 ITMO University, 49 Kronverkskii Ave., St. Petersburg, 197101 Russia; 5 Institute of Materials Research, Helmholtz-Zentrum Geesthacht, Max-Planck-Strasse 1, 21502 Geesthacht, Germany

## Abstract

Super-Planckian near-field radiative heat transfer allows effective heat transfer between a hot and a cold body to increase beyond the limits long known for black bodies. Until present, experimental techniques to measure the radiative heat flow relied on steady-state systems. Here, we present a dynamic measurement approach based on the transient plane source technique, which extracts thermal properties from a temperature transient caused by a step input power function. Using this versatile method, that requires only single sided contact, we measure enhanced radiative conduction up to 16 times higher than the blackbody limit on centimeter sized glass samples without any specialized sample preparation or nanofabrication.

## Introduction

In the far field, the electromagnetic radiation emitted by a hot body is limited to the well-known blackbody distribution originally derived by Planck^1^. However, this theory is only valid when the characteristic dimensions of the body are significantly greater than the wavelength of maximal thermal radiation, ~10 µm at 300 K. When bodies at different temperatures do not satisfy this criterion, then the radiative heat flux may display drastically different characteristics. Particularly, if the separation distance is smaller than the maximum wavelength the radiative heat flux can be orders of magnitude greater than the blackbody limit, a phenomenon known as near-field radiative heat transfer (NFRHT)^2,3^. This results from the contribution of tunneling (evanescent) thermal photons associated with large tangential wave numbers across the vacuum gap separating the bodies. The smaller the separation distance is, the greater the contribution of these decaying modes and, consequently, the higher the heat flux^2–4^.

Given its importance as a generalization of Kirchhoff law of thermal radiation, NFRHT has garnered considerable scientific interest, spanning computational aspects^4–6^, the effect of surface^7,8^ and hyperbolic modes^9–13^, and potential applications^14–18^. A number of experiments demonstrating NFRHT in different geometries have also been reported, each with their own merits and drawbacks. Tip-plane^19–27^ and sphere-plane^28–36^ geometries allow for surface mapping and precise distance control, but suffer from complicated data analysis. Plane-plane experiments offer the advantage of simple interpretation and large, easily detectable, heat fluxes^14,37–47^, however, these experiments are complicated by the need to create uniform sub-micrometer gaps. Other geometries combine advantages of these geometries, but suffer from their own challenges, such as elaborate microfabrication of samples^48,49^.

While the details of these experiments are widely varied, they are tied by a single, dominant, operational paradigm: One side is heated with constant power, and the other is kept at constant lower temperature. The temperatures on hot and cold sides, along with heat power flux, are then measured once the system has reached a steady state (stationary), and from these quantities the near-field heat conductance – radiative heat power flux normalized to the temperature difference – is determined. That is, these experiments are all performed under stationary (steady-state) conditions, where by “stationary” we refer to non-time-varying systems. “Steady-state” measurement techniques allow variations at one frequency (and its harmonics), thus include “stationary” measurements, but forbid the measurement of any transient effects. In some of the NFRHT experiments the gap size^14,19,20,24,36,46^ or the heating power^25,36,46^ is modulated to improve sensitivity. Yet even in these cases, measurements are conducted only once steady-state conditions have been achieved, and the measured quantities directly determine the gap conductance.

In theory, this is the simplest (most straight forward and indisputable) method of measuring the power transferred by thermal radiation. However in practice, there are a number of drawbacks:

(I) All other resistances and losses between the sensors must be carefully approximated or eliminated before the gap conductance can be measured.
(II) The sensors and heaters/coolers must be built into the system. This requires sample accessibility from both sides of the gap where thermal heat transfer is taking place, and good contacting. Often, additional micro-/nanofabrication steps are utilized for that.
(III) The time for an object to thermalize is approximately $\sim \,{R}^{2}/\alpha$, with $R$ standing for the largest characteristic length, and α the thermal diffusivity. If a sample has any characteristic dimensions on the centimeter scale then it can quickly become very time consuming – several minutes to several tens of minutes – to wait for thermal transients to decay.

Here, we report a dynamic NFRHT measurement procedure based on the transient plane source (TPS) technique^50,51^ that substantially alleviates these functional concerns. Notably, the approach requires no specialized sample preparation or microfabrication steps. We present experimental data showing the viability of our method using centimeter sized samples consisting of two optical glass disks separated by gaps varying from 7 µm down to 150 nm. This data agrees precisely with expected theoretical values, and marks the highest radiative thermal conductance enhancement, up to 16 times greater than the blackbody limit, reported for such macroscopic samples. Further, our method requires only one side of the sample to be contacted, and provides additional information about the thermal dynamics of the sample not measurable with stationary approaches. Conceivably, it also allows for the resolution of thermal conductance at multiple gaps; and provides a vital step towards analyzing and implementing systems making use of dynamic thermal effects in the near field.

## Methods

### Transient plane source (TPS) technique

The basis of our technique is the transient plane source (TPS) method, commonly used in the determination of bulk thermal parameters. In this approach, a thin disk consisting of a nickel double spiral embedded in Kapton^52,53^ insulation, acts both as a heat source and temperature sensor. To perform a measurement, the sensor/heater is brought into contact with the material under investigation, and a step power input function is applied. The temperature transient is then recorded by measuring the changing resistive load of the sensor/heater which is then a function of the heat conductance of the environment with which the sensor/heater is in contact.

The thermal properties, including any potential boundary effects, of the surrounding material influence the shape of this temperature transient. As each bulk thermal characteristic affects heat transport in a distinct way, the transient contains enough information to simultaneously extract thermal conductivity and diffusivity without knowledge of the specific heat capacity^50,51^. (Our measurements have been conducted using a commercially available TPS 2500 S from Hot Disk AB, and a Kapton sensor/heater of 19.8 mm diameter provided by this same company). To our knowledge, the here presented work is the first extension of the TPS technique to near-field measurements.

### NFRHT measurements

Our near-field measurements are carried out using interferometric quality BK7 and fused silica glass disks with 20 mm diameter and 5 mm height. To create and maintain a gap between the lower disk (substrate) and upper disk (superstrate), monodisperse silica micro-/nanospheres are used as spacers^39^. The gap size is then confirmed by interferometric reflectivity measurements^43,44^. Once prepared, the gap setup is placed onto the TPS sensor/heater using thermal paste (Apiezon L Grease from M&I Materials Ltd) to improve the sensor/heater-substrate contact. (A detailed explanation of the sample specifications and preparation procedures is presented in Supplementary Information S1. Additional information on gap size measurements is presented in Supplementary Information S6).

**Figure 1:**
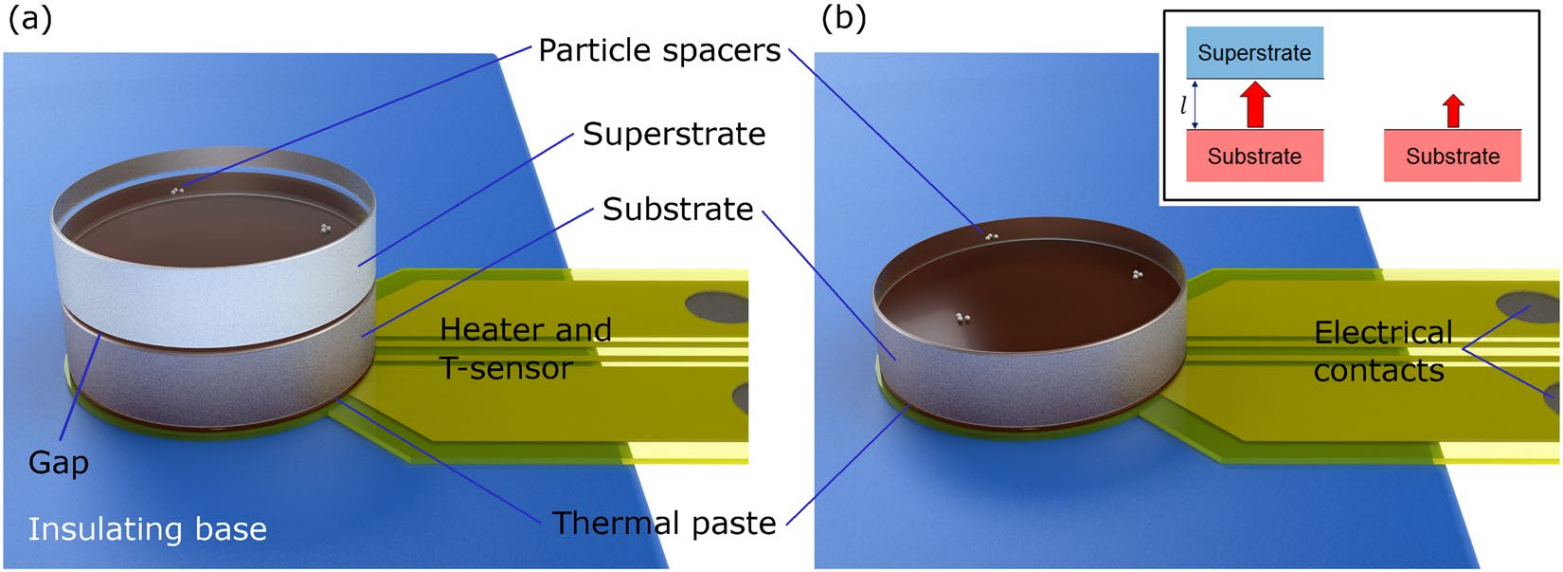
Schematic of experimental setup. (**a**) Two optical glass disks 20 mm in diameter (substrate and superstrate), separated by micro/nano particle spacers, are placed on the TPS sensor/heater. This setup is called the gap sample. To ensure good thermal contact vacuum suitable thermal paste is used. The construction is placed on a thermal insulator with aluminum foil lying in between (not visible) to minimize heat losses due to conduction and radiation. After the transient has been recorded the upper disk is removed, keeping the remaining setup unchanged, and a reference measurement (**b**) is made. This setup is called the reference sample. For easier visualization particles size and TPS sensor/heater thickness are shown not to scale. The inset illustrates the situations at the gap/substrate top. The heat flux from hot substrate to cold superstrate through a gap with small size $l$ (left) is larger than far-field radiation from the substrate top (right). For thermal radiation the glass disks are opaque.

To mitigate both convective and conductive heat flow from the boundaries, the sample and sensor/heater are then placed in a vacuum chamber (reaching a pressure of approximately 10^−5^ mbar). Two measurements are performed. First, the temperature transient of the gap sample is measured without modification. Next, the superstrate is carefully taken away such that the setup remains otherwise unchanged, Fig. 1. This second measurement serves as a reference, thus, this sample is called the reference sample. For both measurements, a power input of 40 mW is applied to the sensor/heater for 640 s. In Fig. 3 two examples of measured gap transients for gap sizes of 315 nm and 2.75 µm are shown for BK7 and for fused silica along with their reference transients. When the glass superstrate is present, near-field radiative heat transfer allows additional heat to escape the substrate, lowering its temperature at constant heating power (Fig. 1 inset). The smaller the gap the stronger this effect is. Note that the transients begin to differ significantly only once the heat front originating from the sensor/heater has reached the upper surface of the substrate, confirming the validity of the reference.

### 1D model and fitting

As the measurement is dynamic, the thermal conductance of the gap cannot be read out directly. Rather, this information must be extracted from the shape of the transient by a fitting procedure. To accomplish this fitting, we utilize two models; a simple 1-dimensional (1D) stack and a more comprehensive, hence complex, 2-dimensional (2D) finite-difference-time-domain (FDTD) gaped cylinder^54,55^.

**Figure 2:**
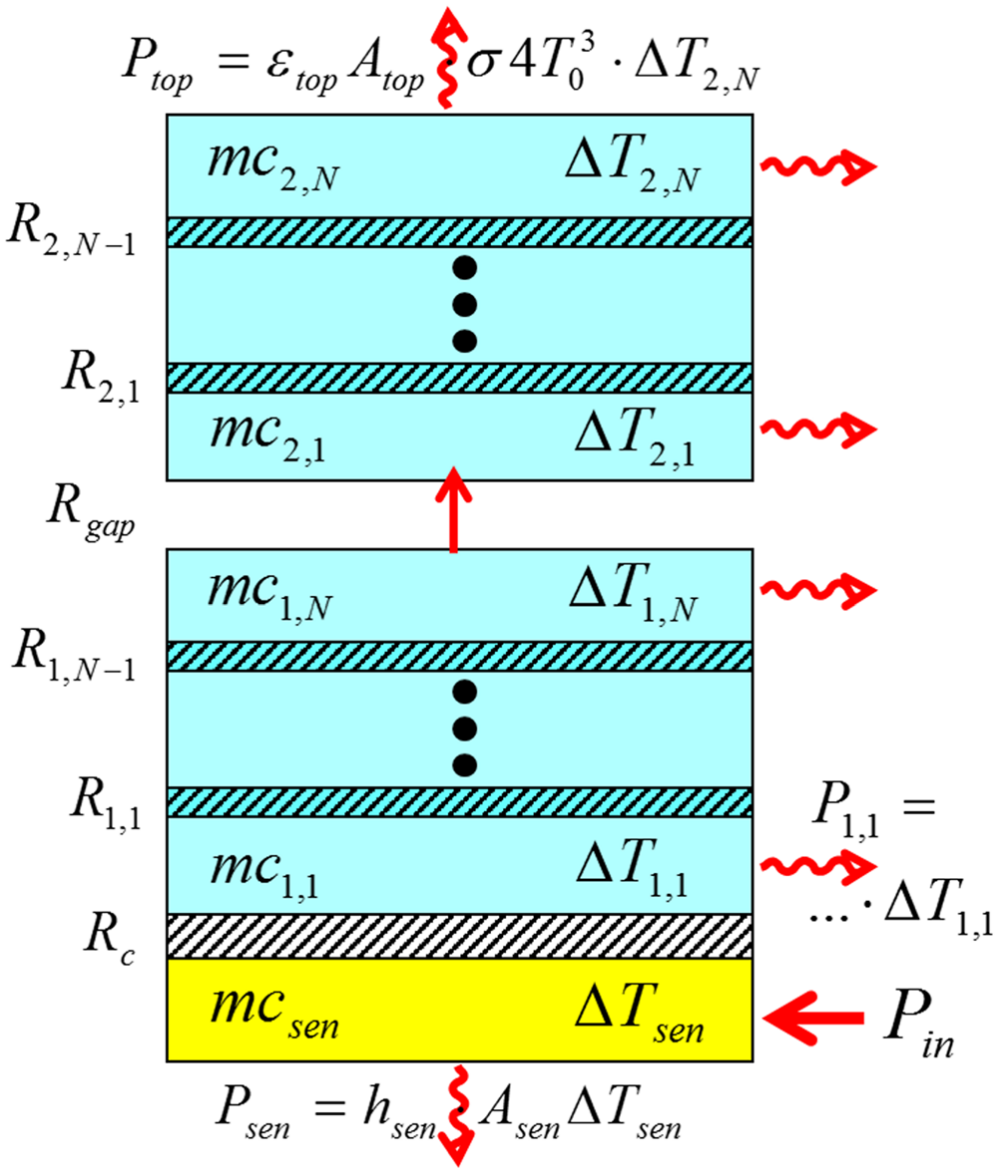
Illustration of the 1D model. The model consists of alternating layers of thermal capacities $mc$ at absolute temperature ${\rm{\Delta }}T+{T}_{0}$ and thermal resistances $R$. The system is initially in thermal equilibrium at temperature ${T}_{0}$ (${\rm{\Delta }}T=0$). At time $t$ = 0 a thermal power of ${P}_{in}$ = 40 mW is applied to the TPS sensor/heater. The system loses energy via conduction and radiation to the bottom ${P}_{sen}$ and via radiation to the top ${P}_{top}$ and the sides ${P}_{1/2,j}$. The sensor/heater is only a few tens of microns thick in total, and so losses to its sides are neglected. As the model is 1-dimensional, we also inherently assume that radiative heat loss from the sides of the sample does not lead to lateral temperature variations. Such variations are later taken into account by our 2D FDTD model, details in Supplementary Information S5.

The framework of the 1D model is illustrated in Fig. 2. The glass disks serving as substrates and superstrates are divided into *N* layers, each with a thermal capacity $m{c}_{1/2,j}$, and a relative temperature ${\rm{\Delta }}{T}_{1/2,j}$ with respect to the thermal equilibrium temperature ${T}_{0}$ = 294 K (±1 K). *N*-1 resistances ${R}_{1/2,j}$ separate these layers, comprising the thermal resistivity of the disks. Additionally, the TPS sensor/heater has a capacity $m{c}_{sen}$ and temperature ${\rm{\Delta }}{T}_{sen}$ and the gap as well as the sensor/heater-substrate contact are represented as resistances ${R}_{gap}$ and ${R}_{c}$. At $t$ = 0 heat with a power of ${P}_{in}$ = 40 mW is applied to the sensor/heater. As time progresses, this input power to the sensor/heater is countered by conductive and radiative losses to the base and radiative losses to free space. We model these additional effects as the conductance ${h}_{sen}$ and emissivities $\varepsilon$, assuming linear dependence on ${\rm{\Delta }}T$. The validity of this assumption rests on the fact that ${\rm{\Delta }}T$ is small compared to ${T}_{0}$, and is one of the reasons a small input power is used. This 1D model provides an analytic solution for the layer temperatures as a function of time, including the sensor/heater temperature. The equations as well as parameter values are given in Supplementary Information S5. Further, the underlying assumptions of 1-dimensionality and linear response are verified by a more comprehensive finite difference model (2D FDTD model).

The fitting procedure proceeds as follows: First, we fit the reference sample measurements; determining the contact resistance $\,{R}_{c}$, top emissivity ${\varepsilon }_{top}$, side emissivity ${\varepsilon }_{side}$ (we assume all side emissivities to be equal ${\varepsilon }_{1/2,j}={\varepsilon }_{side}\,(\forall j)$), and sensor/heater loss conductance ${h}_{sen}$. (${\varepsilon }_{top}$ and ${\varepsilon }_{side}$ are identical for all BK7 references and all fused silica references.) ${R}_{c}$ and ${h}_{sen}$ vary from measurement to measurement as the sensor/heater-substrate contact is intrinsically variable due to varying thickness and morphology of the thermal paste layer used to contact the sensor/heater to the glass disk and due to small differences in the position where the sensor/heater lies on the insulator. The fitted figures are ${\varepsilon }_{top}={\varepsilon }_{side}$ = 0.85 for BK7 and ${\varepsilon }_{top}={\varepsilon }_{side}$ = 0.89 for fused silica. Note that these fits were compared against absorptivity measurements from a Fourier Transform Spectrometer (FTIR) (VERTEX 70 from Bruker), see Figure A2 in Supplementary Information S3. The mean emissivity in the spectral range 5–25 µm is found to be in agreement with the fitted values. To calculate the mean emissivity we assumed the spectral emissivity to equal the measured spectral absorptivity and weighted it with the blackbody spectrum at ${T}_{0}$ = 294 K. Utilizing a Drude-Lorentz model for the glass permittivity (Supplementary Information S3) we checked that increasing the spectral range has no impact on the mean emissivity, consistent with the spectral distribution of blackbody radiation. (In principle, the top and side emissivities can be different due to different roughness.) Fitted ${R}_{c}$ values were found to range from 5 to 11 K/W, while sensor/heater loss conductance ${h}_{sen}$ was found to vary from 0.3 to 5.5 W/(m²K) (below the blackbody heat flux of 5.76 W/(m²K) at 294 K).

**Figure 3:**
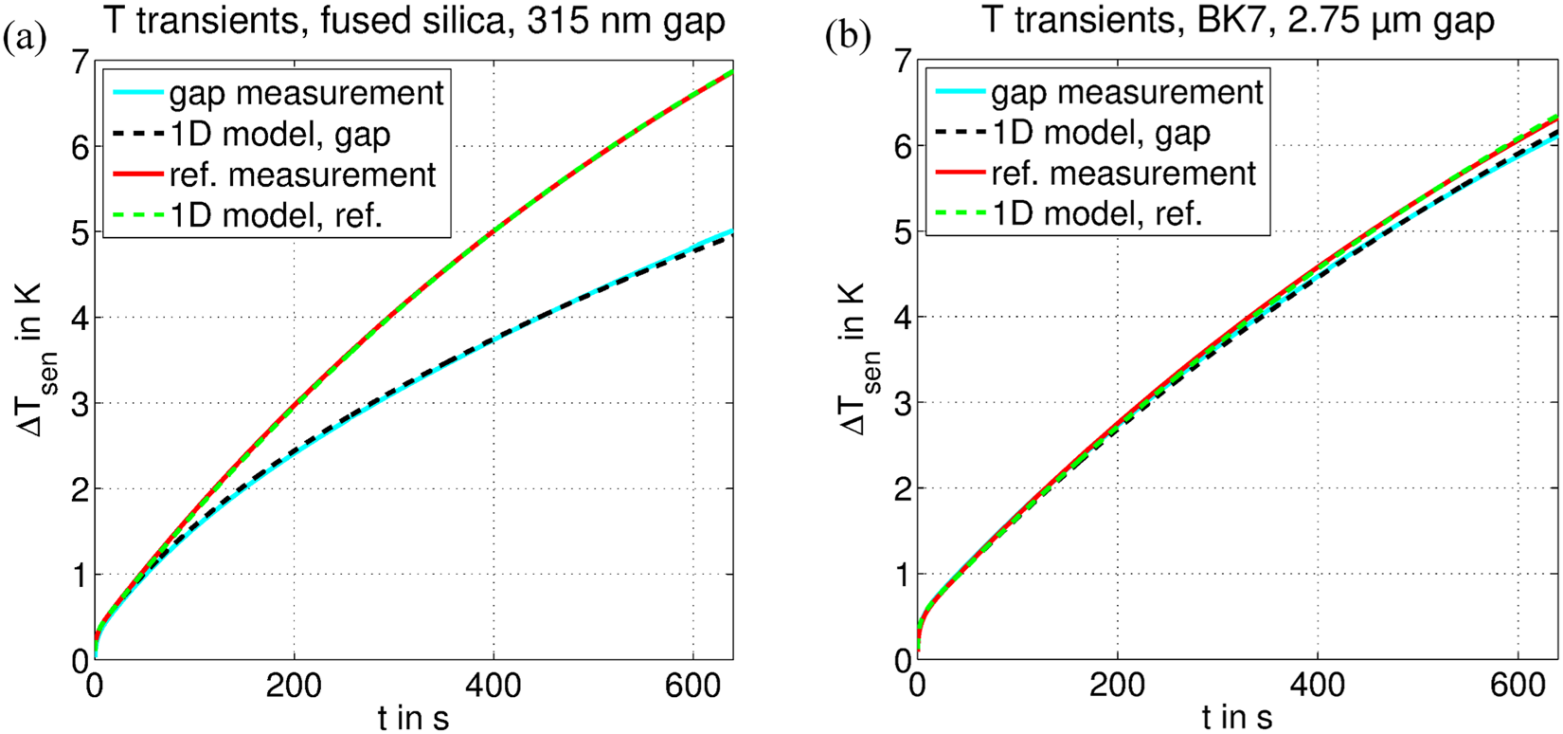
Temperature transients. Sensor/heater temperatures over time for an input power switched on at time *t* = 0 from 0 to 40 mW. Before the power input started the system was in thermal equilibrium at ${T}_{0}$ = 294 K. The samples are composed of (**a**) fused silica glass disks and (**b**) BK7 glass disks. The measured gap is (**a**) 315 nm or (**b**) 2.75 µm, respectively. When near-field radiative heat transfer is present (gap sample measurement) additional heat escapes the substrate, lowering its temperature relative to the reference sample measurement, during which only far-field radiation is present. The smaller the gap the stronger the cooling of the substrate and the larger the separation of gap sample and reference sample transients. The fitted 1D model curves agree very well with the measured ones.

Next, the gap sample measurement is fit using only one parameter, the gap resistance ${R}_{gap}$. Fitted ${R}_{gap}$ values ranged from 35.2 K/W for the smallest gap to 605 K/W for the largest gap. Fitted curves together with the measured ones are shown exemplarily for a 315 nm and a 2.75 µm gap in Fig. 3. Fits and measurements agree very well.

Qualitatively, the transient behavior can be described as follows: In the first few seconds the sensor/heater heats up to few hundred millikelvin above the ambient environment. This increased temperature is necessary for the heat to bridge the contact resistance ${R}_{c}$ and begin to mainly flow into the substrate instead of increasing the sensor/heater temperature. Because the thermal mass of the sensor/heater, $m{c}_{sen}$, is small the temperature increases quickly. The larger ${R}_{c}$ is, the larger this initial temperature increase of the sensor/heater needs to be such that almost all the input power flows into the substrate. Note that as ${R}_{c}\ll 1/({h}_{sen}{A}_{sen})$ sensor/heater losses are irrelevant for these dynamics.

After this initial heating has occurred, the heat front begins moving from the bottom to the top of the substrate. This middle time span captures the bulk thermal properties of the sample. Separation between the two measurements begins as soon as the heat front has reached the top of the substrate. With the thermal properties of the glasses depicted in Supplementary Information S5, we calculated the time to reach the substrate top to be around 10 s. However, we want to emphasize that the separation is a slow and gradual process and thus takes another few tens of seconds until it is clearly visible. For reference sample measurements and gap sample measurements employing large gaps, in which case the upper disk practically keeps its initial temperature, the remaining dynamic is then governed by the sensor/heater and substrate heating up together while losing heat at the sensor/heater, the disk sides and the top/gap. The large thermal mass of the disks makes this dynamic significantly slower. Any changes to the top emissivity or gap conductance alter the shape of this portion of the transient. For smaller gaps, the NFRHT is large enough to allow the superstrate disk to heat up significantly, producing dramatic alterations to the shape of the transient curve.

In principle, all the variables can be obtained directly from the gap measurement without the need for a reference. Following the above description, unique identification is possible as each parameter influence the transient in a different way. E.g. ${R}_{c}$ is proportional to the initial steep temperature increase in the first few seconds, whereas ${R}_{gap}$ start to influence the transient only after some time, the time the two curves in Fig. 3 start to deviate. However, in our particular setup, the sensor/heater losses ${h}_{sen}$ and gap conductance $1/{R}_{gap}$ have a similar effect on the transient; and while differentiation is theoretically possible, due to the delayed influence of the gap conductance vs. the non-delayed influence of the sensor/heater losses, it is not practical. An improved design with a better, e.g. active, thermal insulation may reduce ${h}_{sen}$ to negligibly small values and render the problem void. Similarly, sensor/heater losses ${h}_{sen}$ and side losses ${\varepsilon }_{side}$ have a combined effect, not distinguishable in practice. However, a differentiation is not needed to obtain the gap heat flux. Nevertheless, the two-step gap-reference measurement procedure outlined above provides extra information without introducing new unknowns, and increases both the certainty and accuracy of the fitted results.

Note that the temperature transients in Fig. 3 have not yet reached a steady state. Theoretically, their exponential characteristic prevents the transients to ever reach steady state. However, due to environmental fluctuations, we can say the system is practically in steady state when the temperature is only a few percent away from its steady-state value. Using our 1D model we estimated for the gap setup the time till sensor/heater temperature becomes less than 5% below its steady-state value. The time ranges from 2200 s to 4100 s, depending on the concrete parameters. This coincides with settling times of 30-60 min reported for near-field measurements of centimeter sized samples^40,43^. In any case, our measurement time of 640 s is more than three times shorter than the settling time. An even shorter measurement time can be envisaged as the deviation between gap and reference measurements is visible already after 200 s.

### Data availability

The authors declare that the data supporting the findings of this study are available within the article and its Supplementary Information file. The raw measurement data are available from the corresponding author on reasonable request.

## Results

**Figure 4:**
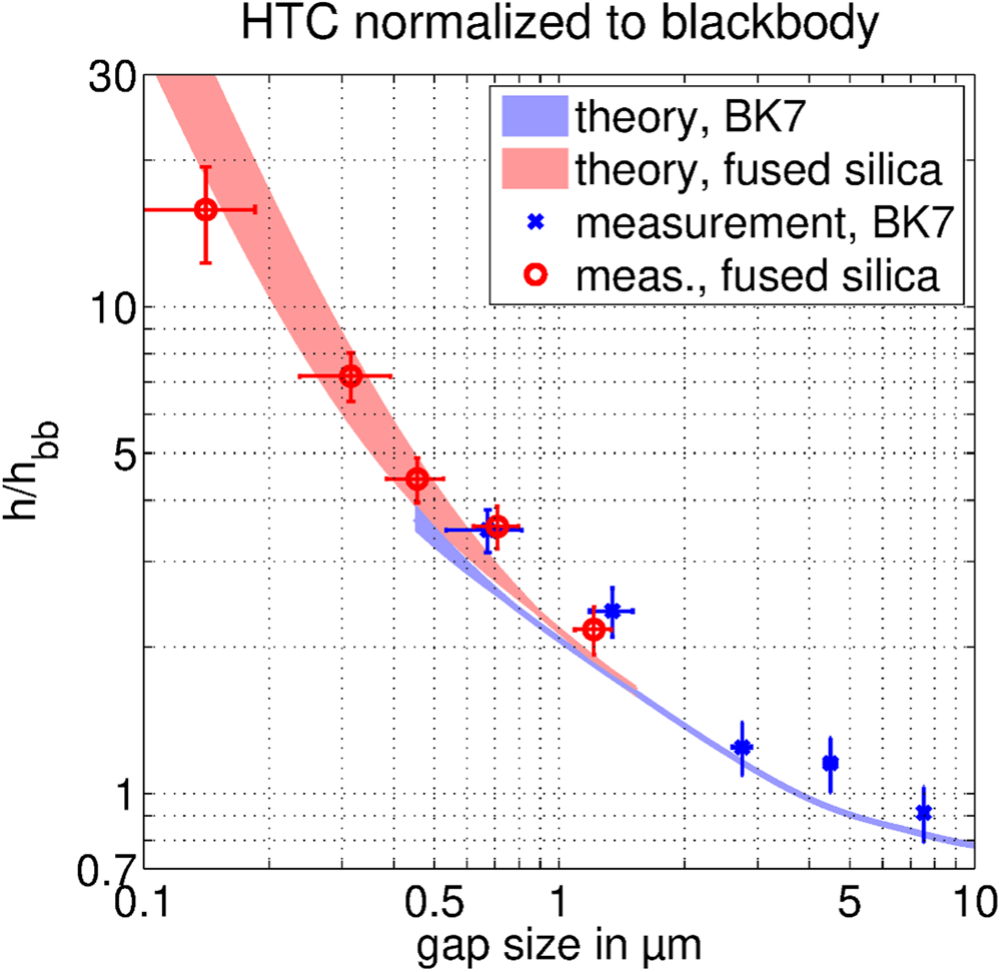
Gap dependent heat flux. Comparison of theoretical and measured radiative heat transfer coefficients (HTC) $h$ versus gap size. The figure shows the heat transfer coefficient (HTC) $h$, normalized by the blackbody result ${h}_{bb}$. Colored areas represent the possible range of theoretical heat flux due to uncertainties in the samples’ optical properties. The slightly higher measured values for larger gaps are attributed to conduction through the spheres used as spacers for the gap. Gap size error margins (horizontal) are small for large gaps and thus hardly visible underneath the symbols. Overall strong agreement is observed between theory and experiment. The 16 times enhancement seen for the 150 nm gap sample is presently the largest recorded for centimeter sized samples at ambient temperatures.

Figure 4 shows the heat transfer coefficient (HTC) – the thermal power flow across the gap per unit cross sectional area per unit temperature difference – normalized to the blackbody HTC ${h}_{bb}$ = 5.76 W/(m²·K) (at 294 K) for different gap sizes. Each of the points corresponds to one measurement. The error margin in the horizontal axis corresponds to the error in the determination of the gap size (see Supplementary Material S6). The relative gap size error increases for the smaller gaps. The vertical error margins in Fig. 4 represent our measurement inaccuracy and combine the effects from fitting procedure and parameter changes between gap and reference measurements. The uncertainty for each measurement is the quadrature of an up to 8% fitting uncertainty, and a 10% variation accounting for possible changes in the sensor/heater losses between gap and reference measurement. The individual contributions are not summed up but the square root of the sum of their squares is taken (root sum square). This leads to an estimated inaccuracy of up to ±13%.

To confirm the reproducibility of the measured results we repeated measurements for the fused silica optical flats with 1.3 µm gap. In addition, the number of deposited microspheres (spacers) was systematically decreased in five measurements to determine the influence of the heat conduction through the microspheres on the apparent radiative HTC. Also, the measurement with the smallest number of particles was repeated four times to check the reproducibility. For additional details see Supplementary Information S2. These series of measurements exhibited no clear trend of the heat flux and a standard deviation of 7.5% which is within the measurement accuracy.

For the 150 nm gap the resistance between the substrate and superstrate becomes comparable to the disks’ inner resistance. Consequently, the influence of this variable on the transient is weaker than for larger gaps and the variation accounting for possible changes in sensor/heater losses becomes 20% instead of 10%. Additional details are provided in Supplementary Information S7.

The theoretical areas are calculated using the well-known integral expression for the near-field heat flux between two half spaces first established by Polder and van Hove.^3–5^. As the optical properties of our samples are known only within a given margin of uncertainty the theoretical HTC is represented as an area, rather than single line. The optical properties used are examined in Supplementary Information S3, and further details of the calculation presented in Supplementary Information S4.

The measured HTCs are observed to be in good agreement with the theoretically predicted range of NFRHT. With decreasing gap size the HCT predictably increases due to the increasing contribution of evanescent modes, consistent with Rytovian electrodynamics^2–4,7^. Besides, confirming our dynamic measurement approach, the measurements themselves are notable. The recorded near-field heat flux ~16 times above the blackbody for the 150 nm gap is presently the largest recorded radiative enhancement for centimeter sized samples at ambient temperatures. At the smallest gap size the surface modes^7,8^, supported by our fused silica samples, create a significantly larger enhancement of the heat flux as compared to purely dielectric samples^47^. We also note that the creation of a gap as small as 150 nm over macroscopic cross sections has previously been reported in only one other experiment^47^, where only an 8.4 heat flux enhancement over blackbody was demonstrated.

For larger gaps a small systematic overestimation of the HTC is observed. We attribute this ≈20% offset to the solid conduction through the supporting microspheres (Supplementary Information S2). Their contribution is reduced at smaller gap sizes as the solid conductance approaches a constant value and the radiative conductance approaches square of inverse gap size dependency. However, it should be noted that the HTC is unequivocally dominated by radiation for all gap sizes (Supplementary Information S2).

## Discussion

The additional information contained in any dynamic or transient approach is both a strength and weakness compared to steady-state measurement techniques. Fundamentally, the measurement is not direct, and the desired parameters are only obtained by iteratively solving an inverse problem. Even for a relatively simple system, like the one we have considered, this introduces the need to carefully consider issues like numerical accuracy, initial values for system parameters, and generally increases the complexity of data analysis.

In return, the strict conditions that are required for a stationary measurement are relaxed. Steady-state measurements require at least three sensors: one sensor for measuring the temperature on each side of the gap, and additional flux sensor for measuring either the heating or cooling power. (Note that the flux sensor may be incorporated as part of the temperature sensor as it is in the TPS method). Crucially, there must be only one undetermined thermal resistance between these sensors. If it is not possible to isolate the NFRHT in this way, then prior to measurement all other heat conduction pathways must be properly modeled before any measurements can be made. Comparatively, in a transient approach the influence of a resistance varies relative to its position from the sensor/heater. The further away from the sensor/heater it is, the later its influence starts. This segregation of effects based on their temporal influence range has three primary benefits. First, it allows the gap resistance to be distinguished from other (parasitic) resistances in the system from collected data (a posteriori). Second, it allows the sensor/heater to be moved away from gap, separating and protecting the gap which is probed from (influences of) the measuring setup. Third, it allows for the simultaneous determination of solid state thermal characteristics that must be assumed in steady-state measurements, and would allow for more than one gap resistance to be determined in a multi gap setup^56^. As mentioned in the introduction, there may also be significant differences in the time needed to perform an individual measurement. For a dynamic measurement the characteristic duration is determined by the distance that must be traveled by the thermal wave front before the influence of the unknown parameter begins to affect the shape of the transient.

In some experiments, to isolate the NFRHT from other parasitic heat fluxes, the gap size has been modulated with a constant frequency and only the heat flux varying with the same frequency has been analyzed^14,19,20,24,36,46^. Further, analogues of 3ω measuring method have also been pursued^25,36,46^. These are certainly counterparts to the reported technique, but they are not dynamic in sense that measurement remains steady-state. At present, no analogue of the dynamic laser flash, transient line, or plane source methods used routinely in measuring solid thermal properties exists for NFRHT measurements.

For academic settings, particularly if all the other thermal characteristics of a given sample are known, these advantages are clearly minimized. Generally, samples are very small and can be carefully designed with multiple micro/nano-fabrication steps. Nevertheless, as NFRHT technologies develop it is highly likely that this will not remain the case. Under even slightly different circumstances, the comparative ability of a dynamic approach to quickly and reliably characterize a variety of samples and reduce design complexity could become critically important. (These considerations extend to any transient technique, and the method we have presented here could, for example, be used in a laser flash system.) It is also worth noting that dynamic measurements are a vital step towards analyzing and implementing systems which make use of dynamic effects, which, by definition, cannot be investigated by steady-state measurements.

## Summary

In summary we have developed a new, dynamic near-field radiative heat transfer measuring method, the first of its kind for this application. Using gap and reference sample types employing two different glass materials and gaps varying from 7 μm to 150 nm we have experimentally confirmed the ability of the approach to accurately measure near-field thermal radiative heat transfer. For the 150 nm gap the measured heat flux is as large as ~16 times above the blackbody. Advantages of this method include simple and precise referencing, the lack of required micro/nano fabrication, and the ability to temporally resolve the influence of various thermal parameters; which helps to distinguish the gap conduction from other effects like interface resistances. The method can also easily be modified and extended. Our results bring to light so far neglected transient measurement techniques, and open the door for the investigation of dynamic radiative effects.

## Electronic supplementary material

Supplementary Information
