# Supplementary material for: Dynamic measurement of near-field radiative heat transfer
Source: Sci Rep. 2017 Oct 24;7:13916. doi: 10.1038/s41598-017-14242-x (PMC5655434; doi:10.1038/s41598-017-14242-x)
Supplement: Supplementary file 1 — Supplementary Information [file 41598_2017_14242_MOESM1_ESM.pdf]

Supplementary Information: Dynamic measurement of near-field radiative heat transfer

S. Lang,[1,*] G. Sharma,[1] S. Molesky,[2] P. U. Kränzien,[1] T. Jalas,[1] Z. Jacob,[2,3] A. Yu. Petrov,[1,4] and M. Eich[1,5]
[1]Institute of Optical and Electronic Materials, Hamburg University of Technology, Eissendorfer Strasse 38, 21073 Hamburg, Germany
[2]University of Alberta, Department of Electrical and Computer Engineering, 9107 - 116 Street, T6G 2V4, Edmonton, Canada
[3]Birck Nanotechnology Center, School of Electrical and Computer Engineering, Purdue University, West Lafayette, IN 47906, USA
[4]ITMO University, 49 Kronverkskii Ave., 197101, St. Petersburg, Russia
[5]Institute of Materials Research, Helmholtz-Zentrum Geesthacht, Max-Planck-Strasse 1, 21502 Geesthacht, Germany

S1: Sample preparation

For our near-field measurements we have used two disks (double surface polished optical flats from Laser 2000) with 20 mm diameter and 5 mm thickness as samples. Two types of optical flats were tested: BK7 glass with a specified surface flatness of λ/10 and fused silica glass with λ/20 peak-to-valley flatness for smaller gaps (λ = 633 nm). The gap between the two flats is maintained by silica spheres serving as spacers, similar to Ref. 1. The particles (monodisperse particles for research purposes from microParticles GmbH) with mean diameters of 7.38 µm, 4.64 µm, 2.79 µm, 1.3 µm, 755 nm, 500 nm, 304 nm, 143 nm and polydispersity less than 5% ensure uniform gaps. Silica is chosen for its good mechanical and thermal stability.

Gaps were created using the following procedure: First, the glass disks are thoroughly cleaned. After ultrasonication in acetone, and then isopropanol, the flats are rinsed with deionized water. Next, the disks are placed in a 60°C bath of water and 2% glass cleaning concentrate (Hellmanex III from Hellma Analytics) and rinsed again. Finally, the disks are

* Correspondence and requests for materials should be addressed to S. L. (email: slawa.lang@tuhh.de)

blown dry with nitrogen. An aqueous suspension is then prepared with the micro particles such that in statistical average three 1 µl drops will contain the desired amount of particles. Three such drops are placed on one disk (the substrate). Using a hot plate the water then is evaporated leaving only the particles on the upper surface. Finally, the second disk (the superstrate) is carefully placed on top of the substrate. The particles cover approximately 10^{-3}% of the ≈314 mm² gap area. Table A1 in Supplementary Information S2 presents the expected number of particles for each measurement. The actual number is subject to statistical variations. Assuming bulk mechanical properties, the deformation of the spheres is calculated to be less than 1%. Even with reduced mechanical properties, as expected for untreated as-synthesized particles[2], sphere deformation is likely limited to a few percent of the radius. The smaller, 300 nm and 150 nm gaps pose a particular challenge for realization and measurement. Although the general approach is the same as for larger gaps, additional measures must have been taken which are listed in Supplementary Information S7.

Note that the substrate-gap-superstrate setup must be contacted only on one side. Thus, the spacer concentration can be reduced compared to conventional steady-state techniques as the upper load is less and the probability to destroy the spacers during setting up is lower. Additionally, the open upper disk allows e.g. simple, optical in-situ gap measurements without access difficulties[3,4].

Gap sizes were measured using reflectivity data versus wavelength obtained from a UV/Vis/NIR spectrometer (LAMBDA 1050 spectrophotometer + URA module from PerkinElmer). The periodicity of Fabry-Pérot interference in this data allows the gap size to be determined to high precision[3,4]. Non-uniform gaps can be easily identified by the

disappearance of the Fabry-Pérot reflectivity characteristic and the appearance of color line patterns. Additional information is provided in Supplementary Information S6.

After confirming the gap size we mount the samples on the TPS sensor/heater. Figure 1 in the main text shows the near-field measurement setup. To improve the thermal contact 1 ml of a vacuum suitable thermal paste (Apiezon L Grease from M&I Materials Ltd) is applied between sensor/heater and substrate. The setup is lying on a thermal insulator (Vacupor from Morgan Advanced Materials Porextherm, conductivity at 1 mbar ≈ 5 mW/(m·K)) and a piece of aluminum foil is placed between sensor/heater and insulator to reduce thermal losses via conduction and radiation. The glass samples lie freely on the sensor/heater, with only the upper disk touching two nylon screws of a sample holder. (Without the holder the upper disk would slide away as friction is extremely low.) The thermal conduction through the screws adds to the side radiation losses. But since nylon is low conducting and due to the very small contact force the contact resistance is high, the effect of the screws is assumed to be negligible.

S2: Number of spacers and their contribution to the heat flux

To create and maintain the vacuum gaps between glass disks we utilize silica particles as spacers. Although this is a rather simple and stable method, it has the disadvantage of introducing solid thermal conduction through the spacers which adds up to the radiative heat flux. This effect is estimated in this section. Table A1 displays the expected numbers of particles used for the experiments. The actual number is subject to statistical variations. The particle coverage of the ≈314 mm² large gap area is slightly below 10^{-5}. Since the silica disks are a bit lighter than the BK7 ones less particles are used when working with the silica disks, proportional to the sample weights. An upper limit on the solid conduction through the

particles is obtained assuming cylinders instead of spheres with same diameter and neglecting contact resistances. The conduction through the cylinders is given in Table A1 in absolute values as well as normalized to the theoretical radiative heat flux. Details on the calculation of the thermal radiation are provided in Supplementary Information S4 and S3. Part (a) of Table A1 shows the data of the measurements presented in the main text in Figure 4. Part (b) shows the data of the repetitive measurements with a 1.3 μm gap. Besides varying the number of particles we also performed four measurement with 400 particles to check not only the influence of particles and but also the reproducibility of our measurements.

(a) **Type**	**Nominal gap size: $d_{particle}$ [μm]**	**Number of particles: $N_{particle}$**	**Surface coverage:**	**Conduction through particles assuming cylinders [$\mathrm{W}/\mathrm{m^2K}$]**	**Theoretical radiation [$\mathrm{W}/\mathrm{m^2K}$]**	**Conduction / radiation**
BK7	7.38	56	$8\cdot10^{-6}$	1.35	4.75	28%
	4.64	142	$8\cdot10^{-6}$	2.15	5.33	40%
	2.79	394	$8\cdot10^{-6}$	3.57	6.63	54%
	1.3	1814	$8\cdot10^{-6}$	7.66	10.2	75%
	0.755	5377	$8\cdot10^{-6}$	13.19	14.27	92%
Fused silica	1.3	1601	$7\cdot10^{-6}$	6.76	10.42	65%
	0.755	4746	$7\cdot10^{-6}$	11.65	15.42	76%
	0.5	10822	$7\cdot10^{-6}$	17.59	22.56	78%
	0.304	9758	$2\cdot10^{-6}$	9.64	41.52	23%
	0.143	138250	$7\cdot10^{-6}$	64.25	141.35	45%

(b) Type	Nominal gap size: $d_{particle}$ [µm]	Number of particles: $N_{particle}$	Surface coverage:	Conduction through particles assuming cylinders $[\mathrm{W}/\mathrm{m}^2\mathrm{K}]$	Theoretical radiation $[\mathrm{W}/\mathrm{m}^2\mathrm{K}]$	Conduction / radiation
Fused silica	1.3	1601	$6.8 \cdot 10^{-6}$	6.76	10.42	65%
		1300	$5.5 \cdot 10^{-6}$	5.49		53%
		1000	$4.2 \cdot 10^{-6}$	4.23		41%
		700	$3 \cdot 10^{-6}$	2.96		28%
		400	$1.7 \cdot 10^{-6}$	1.69		16%

TAB. A1. **Number of particles used as spacers and their maximal contribution to the heat flux via solid conduction.** (a) represents the measurements with different gaps. (b) displays the values for the repetitive measurements with a 1.3 µm gap. The given number of particles is the value expected from amount and concentration of the particle suspension used. The actual number is subject to statistical variations. Surface coverage is defined as $N_{particle} d_{particle}^2 / d_{disk}^2$ where the disk diameter is $d_{disk} = 20$ mm. To estimate an upper limit on the heat flux via solid conduction we assumed silica cylinders instead of spheres with conductivity $\lambda_{\mathrm{SiO_2}} = 1.3\ \mathrm{W/(m \cdot K)}$ and neglected contact resistances. Details on the calculation of the radiative heat flux can be found in Supplementary Information S3 and S4.

Although the solid conduction seems relatively high, the cylinder assumption is definitely overestimating the effect. Spheres are conducting less than cylinders due to smaller contact area. Additionally, there are contact resistances at the particle-disk interfaces adding to the

total thermal resistance. And because the particles are not perfectly monodisperse and the disks not perfectly flat, the number of particles actually being in contact with the superstrate is less than the total number. Overall, we can be certain that the thermal conduction through the gap which we measure is dominated by radiation. The effect of solid conduction can be also estimated from heat flux measurements. The solid conduction through spacers is a reasonable explanation for the small, positive offset of the measured, larger gaps HTCs w.r.t. the theoretical, purely radiative HTCs, seen in Figure 4 (main text).

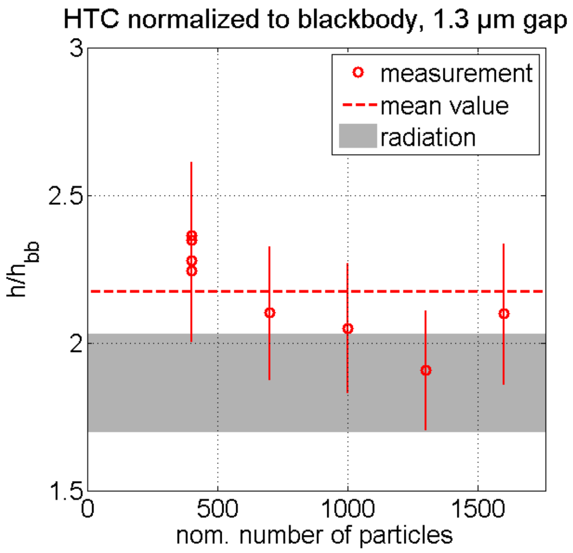

FIG. A1. **Particle dependent heat flux.** Heat transfer coefficient (HTC) h from thermal radiation theory and from experiments versus nominal number of particles for repetitive measurements with fused silica and a 1.3 µm gap. The HTC h is normalized to the HTC between blackbodies h_{bb}. The grey band representing the theoretical radiative HTC includes fluctuations and measurement uncertainties of the gap size, as well as the uncertainty of the optical properties of our fused silica (Supplementary Information S3). The error bars contain the fitting uncertainty (for these measurements up to 5%) and possible changes of sensor/heater losses (leading up to a 10% error). The dashed line is the mean value of the 8 measurements. The standard deviation is ≈7.5%. The slightly higher measured values are probably caused by conduction through the spheres used as spacers for the gap.

The repetitive measurements with decreasing number of spacers, presented in Figure A1, show no clear trend. This, together with the fact that the gap setup is still mechanically stable even with much less particles, indicate that indeed the number of particles in contact with lower and upper samples, or in other word actually holding the upper sample, is less than the total number and not varying too much while we varied the total number. Only these particles increase the heat flux by solid conduction. All in all, the mean gap conductivities of the eight measurements with fused silica and a 1.3 µm gap vary by ± 7.5% around the mean value. This is our measurement reproducibility. The variation is probably mainly due to the measurement inaccuracy and due to real variations of the gap heat flux. The gap heat flux fluctuations come from variations in gap size, and thus in radiative heat flux (≈9%), and from variations of solid heat flux through the spacers. The number of particles in contact with both samples vary statistically, as properties like contact resistances do too. Consequently, the solid conduction through them changes. Further reasons can be small variations in gap uniformity and small variations in equilibrium temperature T_0.

The uncertainty of the fitting procedure is typically around ± 2% but can go up to 8%. Finally, the variation in sensor/heater losses from gap to reference measurement (we estimated a maximum of 10% of blackbody HTC) translates into an error up to 10%. Similar to the standard deviation of the sum of stochastic variables, the individual contributions are not summed up but the square root of the sum of their squares is taken (root sum square). This leads to an inaccuracy of up to ± 13% which we take as our error margin.

The mean gap conductivity of the eight measurements with fused silica and a 1.3 µm gap is offset by ≈20% above the expected value from thermal radiation only. We attribute this offset to solid conductivity through the spacers. The solid conductance is inversely proportional to

the sum of a constant term, representing the contact resistances between spheres and samples, and a term proportional to the gap size, representing the resistance through spheres. At small gap sizes the solid conductance should approach a constant value. Thus, the relative effect of solid conductivity should diminish as soon as the thermal radiation contribution starts to increase at smaller gap sizes, and consequently the relative share of solid conduction in the total heat flux decreases. Exactly this behavior is observed in Figure 4 (main text).

Moreover, the measured HTCs are normalized to the blackbody HTC at the initial equilibrium temperature T_0. In the experiment, the mean temperature at the gap will be fractionally higher, so that the true blackbody normalization factor should correspondingly be slightly larger. The smaller the HTC is, the larger this effect will be. (Note that the normalized theory curves are rather insensitive to changes in T_0.)

S3: Optical properties of BK7 and fused silica glass

Since we measure the radiative heat flux at room temperature most of the thermal radiation is in the mid infrared (MIR), around 10 µm wavelength. To obtain the optical properties of our BK7 and fused silica samples in the MIR, we first looked at literature values for fused silica[5]. The frequency dependent relative permittivity of fused silica can be well described by a Drude-Lorentz model with three resonances

$$\varepsilon(f) = \varepsilon_\infty + \sum_{j=1}^{3} \frac{A_j f_j^2}{f_j^2 - \mathrm{i} f_{col,j} f - f^2}. \tag{A1}$$

The parameters are: $\varepsilon_\infty = 2.01$, $A_1 = 1.071$, $A_2 = 0.113$, $A_3 = 0.77$, $f_1 = 13.52$ THz, $f_2 = 23.83$ THz, $f_3 = 31.92$ THz, $f_{col,1} = 1.43$ THz, $f_{col,2} = 2$ THz, $f_{col,3} = 2$ THz. The permittivity is plotted in Figure A3 (b) (“literature”).

Second, we adjusted the parameters to better fit the reflectivity measured with a Fourier transform infrared spectrometer (FTIR) (VERTEX 70 from Bruker). For BK7 we also assumed the same Drude-Lorentz model because there are no literature data for the MIR. Figure A2 depicts measured and fitted reflectivities which coincide fairly well.

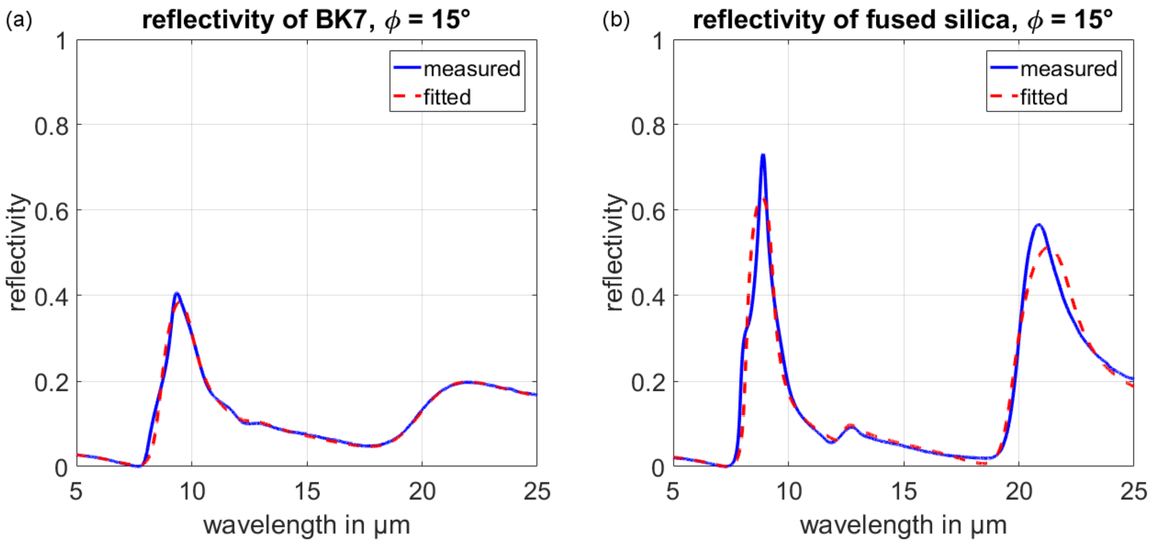

FIG. A2. **Measured and fitted reflectivity** of (a) BK7 and (b) fused silica samples. The angle of incidence is 15°, the light is unpolarized. For the permittivity of BK7 and of fused silica we assumed a Drude-Lorentz model with three resonances causing the three peaks in reflection; the middle peak is very weak.

The parameters obtained from fitting the reflectivity are for BK7: $\varepsilon_\infty = 2.25\ (\pm 0.18)$, $A_1 = 0.796\ (\pm 0.122)$, $A_2 = 0.028$, $A_3 = 0.741\ (\pm 0.076)$, $f_1 = 13.9\ \text{THz}\ (\pm 0.6\ \text{THz})$, $f_2 = 23.8\ \text{THz}$, $f_3 = 30.2\ \text{THz}\ (\pm 0.6\ \text{THz})$, $f_{col,1} = 2.9\ \text{THz}\ (\pm 0.7\ \text{THz})$, $f_{col,2} = 2\ \text{THz}$, $f_{col,3} = 3\ \text{THz}\ (\pm 0.5\ \text{THz})$. And for fused silica: $\varepsilon_\infty = 2.1\ (\pm 0.36)$, $A_1 = 0.805\ (\pm 0.169)$, $A_2 = 0.063$, $A_3 = 0.575\ (\pm 0.134)$, $f_1 = 13.6\ \text{THz}\ (\pm 0.5\ \text{THz})$, $f_2 = 24\ \text{THz}$, $f_3 = 32.3\ \text{THz}\ (\pm 1\ \text{THz})$, $f_{col,1} = 0.8\ \text{THz}\ (\pm 0.5\ \text{THz})$, $f_{col,2} = 1.4\ \text{THz}$, $f_{col,3} = 1.2\ \text{THz}\ (\pm 0.9\ \text{THz})$. The corresponding relative permittivities can be seen in

Figure A3. The values in brackets represent the uncertainty margins of the fits. The second of the three resonances is very weak and reasonable changes in its parameters have practically no effect. For this reason we skip the corresponding uncertainty margins.

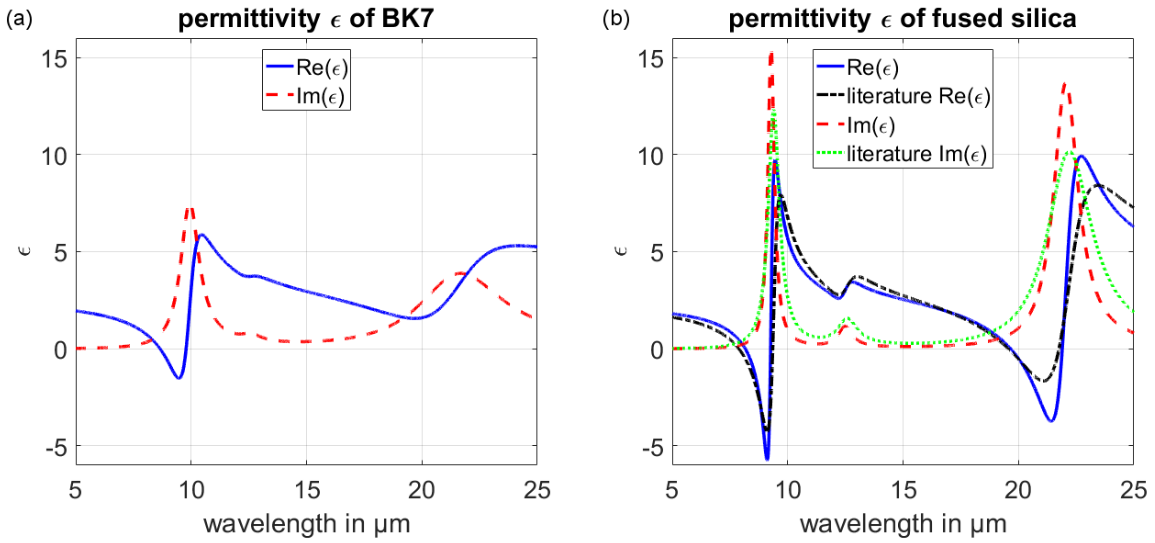

FIG. A3. **Relative permittivity** of (a) BK7 and (b) fused silica obtained from fitting the reflectivity. In both cases a Drude-Lorentz model with three resonances is applied. Fused silica shows the stronger resonances. Additionally, literature values[5] approximated by a Drude-Lorentz model with three resonances are plotted for fused silica. Our measurements indicate that the resonances of our silica disks are stronger than expected. Both glasses have at least one region with negative (real part of) permittivity.

The silica resonances are stronger than expected from literature, and much stronger than the BK7 resonances. However, both materials feature at least one region with negative (real part of) permittivity and thus support surface modes in a spectral range where they significantly contribute to the NFRHT[6,7]. Both theory and measurements exhibit negligibly small transmission. It can therefore not be used to characterize the materials.

S4: Calculation of near-field radiative heat transfer

Knowing the optical properties of the samples, the near-field heat flux between them can be calculated using the integral expressions established by Polder and van Hove[8-10]. Note that since transmission through our samples is zero, we can utilize the simple, two homogeneous half spaces setup for our calculations and don't need to worry about the finite thickness of the disks. The heat transfer coefficient (HTC) – the thermal power flow across the gap per cross section area per temperature difference – is

$$h = \sum_{j=\mathrm{s,p}} \int_0^\infty \frac{d\Theta(\omega,T)}{dT} \iint_{-\infty}^{\infty} \mathrm{T}^j(\omega, k_x, k_y) \frac{dk_x dk_y}{(2\pi)^2} \frac{d\omega}{2\pi}, \quad \text{(A2)}$$

with the energy transmission coefficient

$$\mathrm{T}^j(\omega, k_x, k_y) = \begin{cases} \dfrac{\left(1-\left|r_1^j\right|^2\right)\left(1-\left|r_2^j\right|^2\right)}{\left|1-r_1^j r_2^j \exp(2\mathrm{i}k_z^0 l)\right|^2}, k_\rho < \dfrac{\omega}{c} \\ \dfrac{\mathrm{Im}\left(r_1^j\right)\mathrm{Im}\left(r_2^j\right)\exp(-2|k_z^0| l)}{\left|1-r_1^j r_2^j \exp(-2|k_z^0| l)\right|^2}, k_\rho > \dfrac{\omega}{c} \end{cases}. \quad \text{(A3)}$$

Here $\Theta(\omega,T) = \hbar\omega/(\exp(\hbar\omega/k_B T) - 1)$ is the Bose-Einstein function, T is the mean temperature, $\omega = 2\pi f$ is the angular frequency, $k_\rho = \sqrt{k_x^2 + k_y^2}$ is the wave vector component of the radiation parallel to the interfaces, l is the gap size, c is the speed of light in vacuum, k_B is the Boltzmann constant, $\hbar$ is the reduced Planck constant and j denotes the s- or p-polarization. The transmission coefficient exhibits different expressions for modes propagating ($k_\rho < \omega/c$) or being evanescent ($k_\rho > \omega/c$) inside the vacuum gap. $r_{1/2}^{\mathrm{s}}$ and $r_{1/2}^{\mathrm{p}}$ are the Fresnel coefficients for reflection of s- and p-polarized light at the half spaces 1 and 2. With isotropic half spaces $r_{1/2}^{\mathrm{s}}$ and $r_{1/2}^{\mathrm{p}}$ are given by

$$r_i^{\mathrm{s}} = \frac{k_z^0 - k_z^i}{k_z^0 + k_z^i}, \quad \text{(A4)}$$

$$r_i^{\mathrm{p}} = \frac{\varepsilon_i k_z^0 - \varepsilon_0 k_z^i}{\varepsilon_i k_z^0 + \varepsilon_0 k_z^i}. \quad \text{(A5)}$$

Finally, $k_z^i = \sqrt{\varepsilon_i(\omega/c)^2 - k_\rho^2}$ is the normal component of the wave vector inside medium i which is characterized by relative permittivity ε_i. All media are nonmagnetic. Medium 0 is vacuum ($\varepsilon_0 = 1$). We perform the integrations in (A2) numerically in MATLAB.

The thermal transients with their seconds-range or even minutes-range dynamics are slow and we can utilize the stationary theory of NFRHT.

S5: Additional details on 1D and 2D models

The 1D model described in the main text is solved by considering the heat equation for each individual layer (heat capacity mc). Each equation has the form

$$\frac{d\Delta T}{dt} = \frac{1}{mc}\frac{d\Delta Q}{dt} = \frac{1}{mc}P_{in,total}. \tag{A6}$$

where ΔQ is the thermal energy of the layer relative to the initial equilibrium. For a layer j in the middle of the sample, the total input power is

$$P_{in,total\;1/2,j} = \frac{\Delta T_{1/2,j-1} - \Delta T_{1/2,j}}{R_{1/2,j-1}} + \frac{\Delta T_{1/2,j+1} - \Delta T_{1/2,j}}{R_{1/2,j}} - \varepsilon_{1/2,j}A_{1/2,j}\sigma 4T_0^3\Delta T_{1/2,j}. \tag{A7}$$

Similar expressions can be found for the outer sample layers and the sensor/heater layer. $\sigma \approx 5.670367 \cdot 10^{-8}$ $\mathrm{Wm^{-2}K^{-4}}$ is the Stefan–Boltzmann constant. The equations (A6) for all capacity layers, combined with the expressions for the input power (A7), yield a system of linear differential equations

$$\frac{d}{dt}\overrightarrow{\Delta T}(t) = \mathbf{M}\,\overrightarrow{\Delta T}(t) + \vec{P}_{in}, \tag{A8}$$

with initial condition

$$\overrightarrow{\Delta T}(t=0) = 0. \tag{A9}$$

We collected all temperatures in one vector $\overrightarrow{\Delta T} = \left(\Delta T_{sen}, \Delta T_{1,1}, \dots, \Delta T_{2,N}\right)^T$. The $\vec{P}_{in}$ vector is $\vec{P}_{in} = \left(\frac{P_{in}}{mc_{sen}}, 0, \dots, 0\right)^T$ and the tridiagonal $\mathbf{M}$ matrix has the entries

$$M_{1,1} = -\frac{1+h_{sen}A_{sen}R_c}{R_c mc_{sen}},$$

$$M_{1,2} = \frac{1}{R_c mc_{sen}},$$

$$M_{2,1} = \frac{1}{R_c mc_{1,1}},$$

$$M_{2,2} = -\frac{R_c+R_{1,1}+\varepsilon_{1,1}A_{1,1}\sigma 4T_0^3 R_c R_{1,1}}{R_c R_{1,1} mc_{1,1}},$$

$$M_{2,3} = \frac{1}{R_{1,1} mc_{1,1}},$$

$$M_{j+1,j} = \frac{1}{R_{1,j-1} mc_{1,j}},$$

$$M_{j+1,j+1} = -\frac{R_{1,j-1}+R_{1,j}+\varepsilon_{1,j}A_{1,j}\sigma 4T_0^3 R_{1,j-1} R_{1,j}}{R_{1,j-1} R_{1,j} mc_{1,j}},$$

$$M_{j+1,j+2} = \frac{1}{R_{1,j} mc_{1,j}},$$

$$M_{N+1,N} = \frac{1}{R_{1,N-1} mc_{1,N}},$$

$$M_{N+1,N+1} = -\frac{R_{1,N-1}+R_{gap}+\varepsilon_{1,N}A_{1,N}\sigma 4T_0^3 R_{1,N-1} R_{gap}}{R_{1,N-1} R_{gap} mc_{1,N}},$$

$$M_{N+1,N+2} = \frac{1}{R_{gap} mc_{1,N}},$$

$$M_{N+2,N+1} = \frac{1}{R_{gap} mc_{2,1}},$$

$$M_{N+2,N+2} = -\frac{R_{gap}+R_{2,1}+\varepsilon_{2,1}A_{2,1}\sigma 4T_0^3 R_{gap} R_{2,1}}{R_{gap} R_{2,1} mc_{2,1}},$$

$$M_{N+2,N+3} = \frac{1}{R_{2,1} mc_{2,1}},$$

$$M_{N+j+1,N+j} = \frac{1}{R_{2,j-1} mc_{2,j}},$$

$$M_{N+j+1,N+j+1} = -\frac{R_{2,j-1}+R_{2,j}+\varepsilon_{2,j}A_{2,j}\sigma 4T_0^3 R_{2,j-1} R_{2,j}}{R_{2,j-1} R_{2,j} mc_{2,j}},$$

$$M_{N+j+1,N+j+2} = \frac{1}{R_{2,j} mc_{2,j}},$$

$$M_{2N+1,2N} = \frac{1}{R_{2,N-1} mc_{2,N}},$$

$$M_{2N+1,2N+1} = -\frac{1+(\varepsilon_{2,N}A_{2,N}+\varepsilon_{top}A_{top})\sigma 4T_0^3 R_{2,N-1}}{R_{2,N-1}mc_{2,N}}. \quad \text{(A10)}$$

The solution to this system of linear differential equations is

$$\overrightarrow{\Delta T}(t) = \text{expm}(\mathbf{M}t)\mathbf{M}^{-1}\vec{P}_{in} - \mathbf{M}^{-1}\vec{P}_{in}. \quad \text{(A11)}$$

Here $\text{expm}(\cdot)$ is the matrix exponential. The model and the corresponding equations for the reference measurements with only one glass flat are analog to the gap model.

We divided the total thermal capacity of one glass disk into N = 10 equal capacity layers and the total thermal resistance into $N - 1$ = 9 equal resistance layers. Accordingly, the lateral surface area is also divided into N = 10 equal areas $A_{1/2,j}$. Finer discretization, accompanied by larger N, does not further improve the accuracy of simulation results, however, would increase computation time. Glass disks and TPS sensor/heater have a diameter of 20 mm. The disks are 5 mm thick. One BK7 disk weighs m_{BK7} = 3.92 g and fused silica disk m_{FS} = 3.46 g. For our modeling we employ a specific capacity of c_{BK7} = 0.82 J/(g·K) and a conductivity of λ_{BK7} = 1.11 W/(m·K) for BK7. For fused silica the values are c_{FS} = 0.755 J/(g·K) and λ_{FS} = 1.38 W/(m·K). They are taken from the respective datasheets[11,12] (with the specific capacities slightly modified to improve fitting) and agree with independent measurements (performed with TPS 2500 S from Hot Disk AB). Resistances are calculated via $R_{1/2,j} = 5\text{ mm}/\left((N-1)\lambda \cdot \pi(10\text{ mm})^2\right)$. The sensor/heater capacity is mc_{sen} = 85 mJ/K which is increased above the value provided by sensor/heater manufacturer to account for the thermal paste.

We note that the two step fitting procedure for this model, outlined in the main text, is well justified by the similarity of the reference and gap measurements; which ensures no changes of other parameters between gap and reference measurement. Only the sensor/heater loss

parameter h_{sen} might vary due to the change in weight. We estimated from the largest gap measurement – where gap heat flux and far-field emission are almost identical – that h_{sen} does not change by more than 10% of the blackbody heat flux.

The 1D model makes two major simplifications, the applicability of which must be checked. Namely, the model neglects any radial temperature gradients / heat fluxes, and linearizes emitted radiation with respect to ΔT. To check that the effect of these assumptions is small, we utilized a 2-dimensional (2D) finite-difference time-domain (FDTD) model[13]. A 3rd dimension is not necessary as the system is rotationally symmetric. The 2D model consists of three material domains stacked vertically, with identical diameters but possibly different heights. The lowest material domain represents the TPS sensor/heater. Its diffusivity and conductivity are adjusted to yield a low heat capacity and negligible inner temperature gradients. The upper two domains represent the substrate and superstrate. Separating the three regions are two thermal resistances / conductances representing possible gaps (linear in temperature difference). The lower inter-block resistance determines the contact resistance of the substrate and sensor/heater, and the upper resistance the conductivity of the gap. The reference setup is modelled by adjusting the upper block heights and shorting the upper inter-block resistance, such that the upper two blocks appear as a single domain.

Heat flux is applied uniformly at bottom boundary of the lowest domain. At exterior boundaries, each domain is assumed to radiate based on its emissivity. (The thermal radiation is modelled properly proportional to $(\Delta T + T_0)^4 - T_0^4$ or linearized proportional to $4T_0^3\Delta T$.) Inside the material domains the FDTD scheme is employed following the Crank-Nicholson[13] method in cylindrical coordinates. The cell equations are analogues to those given by Dawson et al.[14] except for the added radiative heat loss at boundary elements.

Thermal transients simulated with the model show only minor differences compared with the 1-dimensional method described in the main text. Figure A4 displays calculated temperature profiles for both the gap and reference sample setups. Two things are observed. First, although small radial changes are visible, the heat is mainly flowing in axial direction, resembling a 1-dimensional situation. Second, the temperature differences between the hotter core and cooler exterior boundary amounts to only few 100 mK even for long simulation times. It means the inner sample resistance overall has no big influence on the thermal dynamics. The reason for this agreement, comes from the nearly 1-dimensional character of the system we have considered. The sensor/heater covers the whole sample cross section, and so heat flows mainly in axial direction.

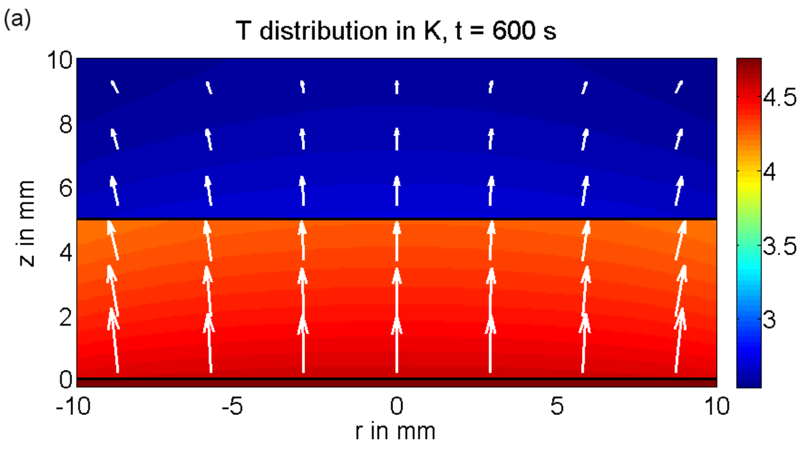

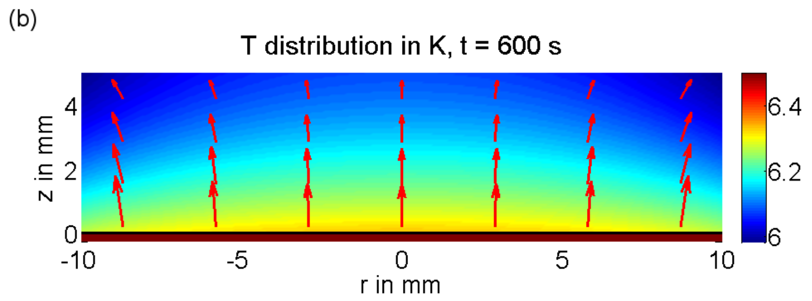

FIG. A4. **Temperature distribution** in (a) the gap sample setup and (b) the reference sample setup at t = 600 s. Both setups are rotationally symmetric and thus, simulations and illustrations show only the axial and radial dimensions. The sensor/heater, seen as the low homogeneously colored slab, is assumed to be 0.25 mm thick and have negligible inner resistance. These 2-dimensional simulations are based on the parameters obtained from the fit of the 300 nm gap measurements. The white and respectively red arrows indicate the direction and strength of the heat flux, which is proportional to the negative temperature gradient. Despite the large side emissivity of ε_{side} = 0.89 the heat flows predominantly in axial direction.

Slightly larger differences are observed in the switch from linear to non-linear emission, but these too are found to be mostly inconsequential. Specifically, the introduction of non-linearity is found to increases the emitted power and, thus, decreases the sensor/heater temperature, Figure A5. This effect is most pronounced at larger temperatures. Yet, overall linearization has also only a weak influence on the transients. Although the 2D model is slightly more accurate, we generate our results with the 1D model because of its exact (analytic) solution and its advantageous computation time.

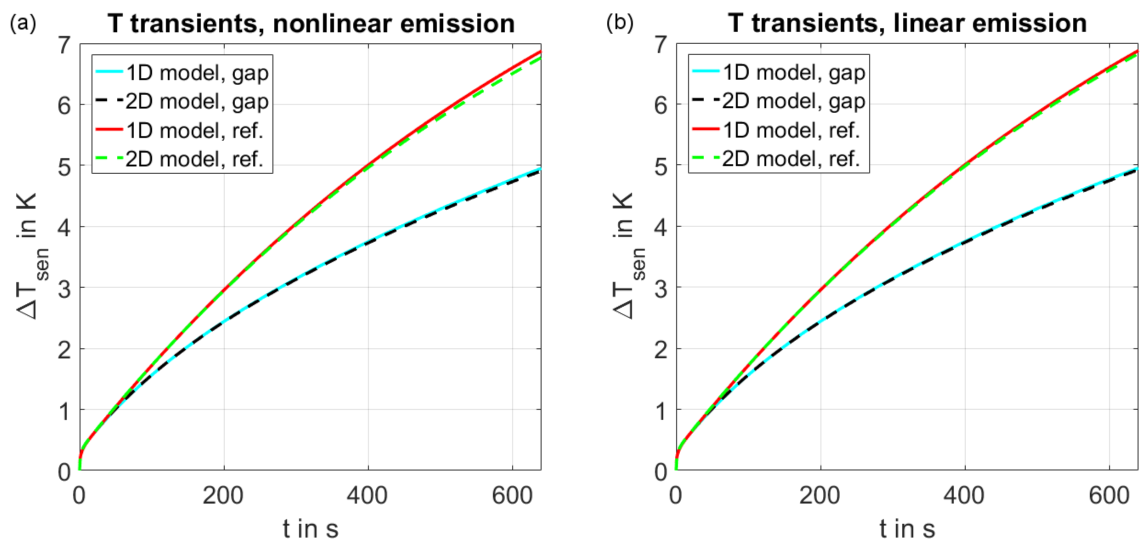

FIG. A5. **Simulated temperature transients.** The simulations are based on the parameters obtained from the fit of the 300 nm gap measurements. For both, 1- and 2-dimensional models, the same parameters are used. The 2D model simulations assume (a) a nonlinear, T^4 emission (including nonlinear sensor/heater losses) and (b) a linearized emission as in the 1D model. In the linearized emission case (b) both models agree very well. Accounting for the radial inner sample resistance practically does not change the transients. The nonlinear emission is stronger than the linearized one leading to slightly weaker heating up, as seen in (a).

S6: Determination of gap size from reflectivity measurements

To verify the existence and determine the size of the gap between the optical disks we measure the reflectivity of the substrate-gap-superstrate setup with a UV/Vis/NIR spectrometer (LAMBDA 1050 spectrophotometer + URA module from PerkinElmer). The reflectivity features result from Fabry-Pérot interference in the gap, with periodicity characteristic of the gap size. The spectral range is limited to the near ultraviolet, visible and near infrared where the glasses are transparent. The concrete wavelength range depends on the gap size and the glass type. Our wavelength resolution is 5 nm.

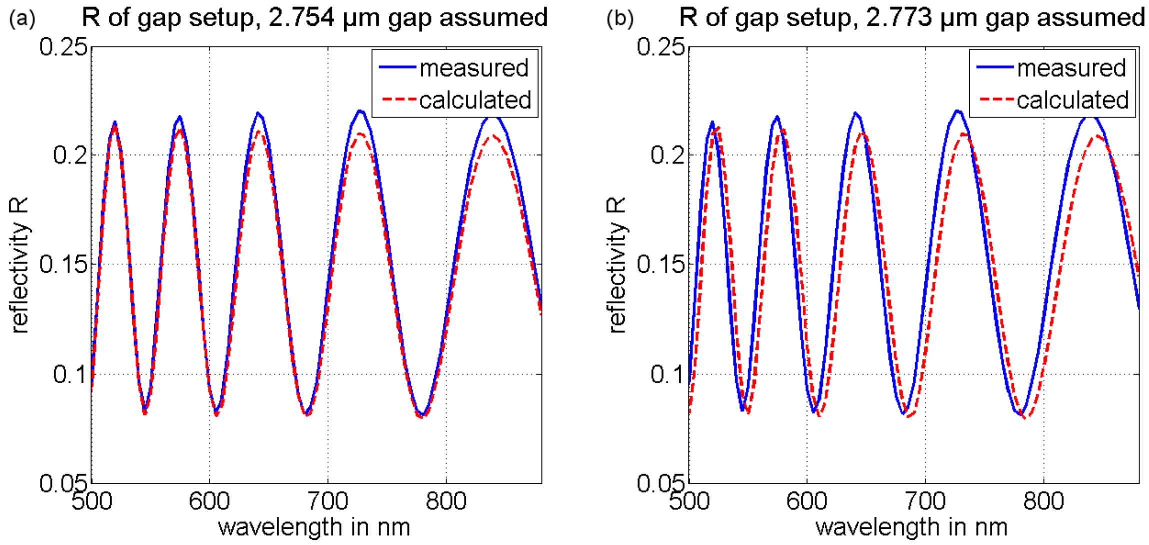

FIG. A6. **Reflectivity of substrate-gap-superstrate setup** with BK7 disks and 2.79 µm particles used as gap spacers. Besides the measured reflectivity also the calculated one is shown assuming (a) a gap size fitting the reflectivity the best and (b) a gap size representing the upper limit of the gap size error margin. The angle of incidence is 8°, the light is unpolarized. The periodicity of the Fabry-Pérot interference in the gap is very sensitive to the gap size leading to very precise gap size determination.

Measured and simulated reflectivity are plotted in Figure A6. The calculation is based on a simple, multiple reflections assumption and given by

$$R_{\text{gap setup}} = R + T^2 R_{\text{gap}} + T^2 R R_{\text{gap}}^2 + T^2 R T_{\text{gap}}^2. \qquad \text{(A12)}$$

R and $T = 1 - R$ are the reflectivity and transmission at the air-glass interfaces. Reflectivity is obtained by taking the absolute values squared of the reflection coefficients provided by equations (A4) and (A5) or the well-known Fresnel equations. Note that reflectivity and transmission of light coming from the air at an angle of incidence of φ = 8° and of light coming from the glass at the respective, refracted angle of incidence are identical. Gap reflectivity and transmission are given by $R_{\text{gap}} = 1 - T_{\text{gap}}$ and the Fabry-Pérot expression

$$T_{\text{gap}} = \frac{T^2}{1 + R^2 - 2R\cos\left(\frac{2\pi}{\lambda} 2l\cos\varphi\right)}. \qquad \text{(A13)}$$

λ is the wavelength and l the gap size.

There is no Fabry-Pérot interference observed from the disk due to the large disk thickness of 5 mm. As directly seen from (A12) the reflectivity of the gap setup takes into account up to two light interactions with the gap. We checked that taking into account more interactions has practically no more effect on the calculated $R_{\mathrm{gap\ setup}}$.

To determine the gap size we first define the fitting error as the sum (over wavelength) of squared differences between measured and calculated reflectivity. The fitted gap size is the one minimizing the error. The uncertainty region is the gap size range which yields errors smaller than two times the minimal error. Figure A6 shows the measured reflectivity for a gap created with 2.79 µm particles and two calculated reflectivities, one assuming the fitted gap size and one assuming the gap size corresponding to the upper uncertainty limit. As the figure depicts, the Fabry-Pérot features are very sensitive to the gap size. Even small deviations from the optimal value make measured and simulated reflectivities different, and therefore, the gap size fitting uncertainty is small. As the gap size is measured at one position an additional deviation of two times λ/10 or λ/20 is always included in the gap uncertainty to account for possible sample non-planarity.

Small variations in the gap size across the 5 x 5 mm² measurement area result in locally different Fabry-Pérot patterns. These different patterns have slightly different periodicities proportional to the inverse of the gap size (remember *R* vs. wavenumber shows a unique period whereas *R* vs. wavelength shows an increasing period). The averaging over the measurement area results in an averaging over the Fabry-Pérot periods and thus, in an averaging over the inverse of the gap size. So (1 / obtained gap size) equals the average of (1 / gap size). In the transition region from far-field to strong near-field heat transfer the heat

flux approximately scales with the inverse of the gap size. Consequently, the average heat flux is the heat flux at average inverse gap size; these are both quantities that we measure[15].

Any significant non-uniformity of the gap, and thus locally shifted interference patterns, results in the disappearance of the pattern in the measurement. Strongly non-uniform gaps can even be identified with the naked eye because they show an interference color line pattern. Furthermore, the upper disk is floating on the lower one because there is almost no friction, especially at larger gaps. If there are locations where the two samples are touching, e.g. since the spacers were destroyed there, we will easily identify them since they lose their floating property. This and the interference color line patterns moreover allow us to make sure that after mounting the samples the gap is still intact.

S7: 300 nm and 150 nm gaps

The deep sub-micrometer small gaps pose a particular challenge. Realizing as well as confirming them is accompanied by additional effort. Here, we provide the important aspects associated with our experiments with 300 nm and 150 nm gaps. 150 nm gaps are the smallest gaps realized in a macroscopic plane-plane geometry up to now[16].

The main issues for realizing small gaps by the spacer method are to overcome clustering of particles and to avoid larger dust / grime particles in the gap. The clustering tends to form multilayers of particles and, therefore, increases the gap by approximately an integer multiple of the particle size. Additionally, this clustering may also induce inclined gaps, if the numbers of layers are unequal between the supporting points. Remember, we place three drops of particle solution on our slides. On the other hand, a large number of spacer particles

is required to mechanically hold the superstrate. Unwanted dust or grime will lead to unpredictable and uncontrollable gaps.

For avoiding the unwanted dust / grime particles in the gap, we performed the cleaning procedure twice. Furthermore, we used fresh samples to ensure there are no surface damages due to previous experiments.

We solved the clustering problem by two separate approaches. First, we reduced the particle concentration, and thus the possibility of cluster formation. For the 300 nm gap the total number of particles is three times less than usual (cf. Table A1). Also, we used three 2.5 µl instead of 1 µl drops which allowed us to further reduce the concentration of particles in the suspension.

Second, for the 150 nm gap we accepted the clustering in the first place and reduced the gap distance by applying a small vertical force while rotating the upper disk, to destroy the clusters but not the particles. For the second approach the gap distance is measured at six different spots, aligned in a cross, with the UV/Vis/NIR spectrometer and a wavelength range from 195 nm to 1500 nm. After the first three spots we turned the sample by 90° horizontally around its center point for measuring the last three spots. With the six spots, we were able to capture the inclination of the plane-plane geometry and provide a gap size averaged over the whole sample area. This is of particular importance for creating the 150 nm gap, since an inclination cannot be identified by a visible interference pattern, as it is the case for gap sizes larger than 300 nm.

The inclination is reduced by applying a small vertical force at the largest gap spot and rotating the superstrate, resulting in a shear force which separates the cluster. Applying a force to destroy the cluster only seems convenient, as long as the amount of particles forming the clusters is low compared to the total amount of particles, because otherwise many

particles would be destroyed, due to the high necessary force. We confirmed this by investigating the particle distribution with a scanning electron microscope (SEM). For that, we prepared object slides with particles on it, under the same conditions as we prepared the sample disks, but omitting the upper disk. As a matter of fact, for the particle concentrations we used, the amount of particles forming a multilayer cluster is low compared to the total amount of particles.

Finally, we want to discuss the uncertainty of the 150 nm gap HTC measurement. It is larger than all other uncertainties. The reason for that is illustrated in Figure A7. The figure shows simulated transients for different gap conductances. Whereas for smaller gap conductances a change by a factor of two significantly alters the temperature transient, for larger gap HTCs above 16 h_{bb} the transient becomes less sensitive and finally saturates to a curve independent of the gap heat flux. This happens when the gap resistance gets in the range of the disk resistance (fused silica disk conductance corresponds to ≈ 48 h_{bb}). It's clear that a very small disk resistance practically doesn't influence the total system resistance and hence the system behavior. With an expected gap heat flux of approx. 18 to 32 times the blackbody value, the 150 nm gap is our only measurement in the saturating region.

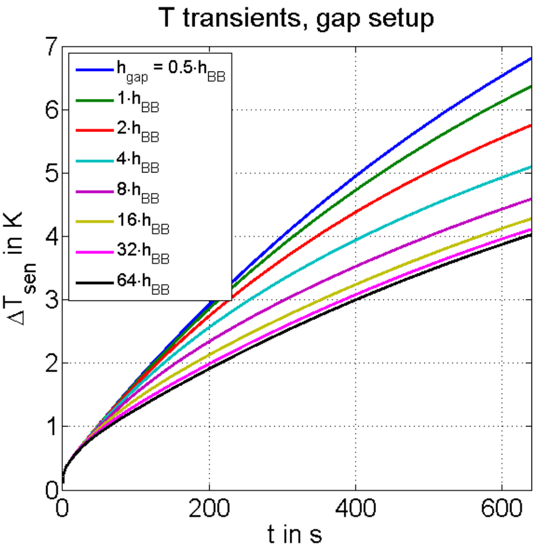

FIG. A7. **Simulated sensor/heater temperatures over time for different gap conductances.** The simulations are based on the fused silica parameters and R_c = 8 K/W, h_{sen} = 2.88 W/(m²·K). The gap conductance is varied from 50% of the HTC between two blackbodies, h_{bb} = 5.76 W/(m²·K), to 64 times the value. Transients of the reference setup are not shown. Whereas for smaller gap conductances a change by a factor of two significantly alters the temperature transient, for larger gap HTCs above 16 h_{bb} the transient becomes less sensitive and finally saturates to a curve independent of the gap heat flux.

This measurement difficulty is not particular to our transient method. In steady state one measures the total resistance. The smaller the portion of gap resistance in total resistance, the less accurate the measurements become, till the gap resistance completely disappears in the resistance measurement inaccuracy. The problem can be avoided by employing thinner and better conducting samples.

References

1. Hu, L., Narayanaswamy, A., Chen, X. & Chen, G. Near-field thermal radiation between two closely spaced glass plates exceeding Planck's blackbody radiation law. *Appl. Phys. Lett.* **92,** 133106 (2008).
2. Romeis, S. *et al.* In Situ Deformation and Breakage of Silica Particles Inside a SEM. *Procedia Eng.* **102**, 201–210 (2015).
3. Ijiro, T. & Yamada, N. Near-field radiative heat transfer between two parallel SiO_2 plates with and without microcavities. *Appl. Phys. Lett.* **106,** 023103 (2015).
4. Ito, K., Miura, A., Iizuka, H. & Toshiyoshi, H. Parallel-plate submicron gap formed by micromachined low-density pillars for near-field radiative heat transfer. *Appl. Phys. Lett.* **106,** 083504 (2015).
5. Palik, E. D. *Handbook of Optical Constants of Solids* (Academic Press, 1985).
6. Joulain, K., Mulet, J.-P., Marquier, F., Carminati, R. & Greffet, J.-J. Surface electromagnetic waves thermally excited: Radiative heat transfer, coherence properties and Casimir forces revisited in the near field. *Surf. Sci. Rep.* **57,** 59–112 (2005).
7. Basu, S. & Zhang, Z. M. Ultrasmall penetration depth in nanoscale thermal radiation. *Appl. Phys. Lett.* **95,** 133104 (2009).
8. Polder, D. & van Hove, M. Theory of Radiative Heat Transfer between Closely Spaced Bodies. *Phys. Rev. B* **4,** 3303–3314 (1971).
9. Park, K. & Zhang, Z. Fundamentals and applications of near-field radiative energy transfer. *Front. Heat Mass Transfer* **4,** 013001 (2013).
10. Biehs, S.-A., Rousseau, E. & Greffet, J.-J. Mesoscopic Description of Radiative Heat Transfer at the Nanoscale. *Phys. Rev. Lett.* **105,** 234301 (2010).
11. "Optical Glass - Collection Datasheets" from SCHOTT AG, downloadable at www.schott.com (2017).
12. "Data sheet: Fused silica for Optics - Data and Properties" from Heraeus Quarzglas GmbH & Co. KG, downloadable at www.heraeus.com (2017).
13. Crank, J. & Nicolson, P. A practical method for numerical evaluation of solutions of partial differential equations of the heat-conduction type. *Math. Proc. Cambridge Philos. Soc* **43,** 50–67 (1947).
14. Dawson, C. N., Du, Q. & Dupont, T. F. A finite difference domain decomposition algorithm for numerical solution of the heat equation. *Math. Comp.* **57,** 63–71 (1991).
15. Lim, M., Lee, S. S. & Lee, B. J. Near-field thermal radiation between doped silicon plates at nanoscale gaps. *Phys. Rev. B* **91,** 195136 (2015).
16. Bernardi, M. P., Milovich, D. & Francoeur, M. Radiative heat transfer exceeding the blackbody limit between macroscale planar surfaces separated by a nanosize vacuum gap. *Nat. Commun.* **7,** 12900 (2016).
